# Experiencing anesthesia and surgery early in life impairs cognitive and behavioral development

**DOI:** 10.3389/fnins.2024.1406172

**Published:** 2024-07-24

**Authors:** Xuqin Jia, Siyou Tan, Yinying Qin, Yi Wei, Yage Jiang, Sining Pan, Chunlai Li, Jing Chen, Tianxiao Liu, Yubo Xie

**Affiliations:** ^1^Department of Anesthesiology, The First Affiliated Hospital of Guangxi Medical University, Nanning, China; ^2^Guangxi Key Laboratory of Enhanced Recovery After Surgery for Gastrointestinal Cancer, The First Affiliated Hospital of Guangxi Medical University, Nanning, China

**Keywords:** surgery, child, cognition, behavioral development, anesthesia

## Abstract

**Background:**

The impact of anesthesia and surgery on neurocognitive and behavioral development in infants and children remains inadequately understood.

**Objective:**

To investigate the impact of early-life exposure to general anesthesia and surgery on cognitive and behavioral development.

**Methods and materials:**

Children aged 0–3 years who underwent general anesthesia and surgical procedures between 2012 and 2015 were included. The cognitive and behavioral development of these children at ages 4–6 years was assessed. Age-, race-, and gender-matched children from the same geographic region, who did not undergo general anesthesia or surgery, served as the control group. The Wechsler Preschool Primary Scale of Intelligence, Fourth Edition (WPPSI-IV) was used to evaluate children’s total intelligence quotient (FSIQ) and specific cognitive domains. The Gesell Development Schedules (GSCH) and Child Behavior Checklist (CBCL) were employed to assess behavioral and personality development. Additionally, the study analyzed the effects of various factors including anesthesia drugs, surgery duration, number of surgeries, age, weight, ethnicity, and gender on postoperative neurocognitive and behavioral outcomes.

**Results:**

The study included 447 children with anesthesia/surgical exposure (AS) and 459 children in the control group. Analysis of cognitive and behavioral development showed a significant difference in the working memory index (WMI) between the AS and control groups (*p* < 0.05). Exploratory findings indicated that children administered remifentanil exhibited lower developmental quotient (DQ) values, whereas those given fentanyl showed higher (worse) Child Behavior Checklist (CBCL) total scores. Moreover, increased anesthesia/surgical exposures, younger age and lower body weight at exposure, and longer surgery durations were associated with cognitive and behavioral developmental challenges.

**Conclusion:**

This study examined the impact of early-life exposure to surgery and anesthesia on postoperative cognitive and behavioral development. Findings indicate that higher frequency of exposure to surgery and anesthesia, younger age, and lower body weight at exposure could negatively influence cognitive and behavioral development. Furthermore, variations in the effects of different anesthetics on behavior and cognition were observed. Caution is advised regarding the use of opioid analgesics such as remifentanil and fentanyl for more rigorous clinical applications.

## 1 Introduction

The impact of anesthesia and surgery on neurocognitive function and behavioral development in infants and young children remains a topic of ongoing concern, without definitive consensus. The US Food and Drug Administration (USFDA) has cautioned against the potential risk of anesthetic neurotoxicity in pediatric patients under 3 years of age, identifying this period as a critical “vulnerable window” for synaptogenesis (Clausen et al., 2018; Ing et al., 2022). Growth and developmental processes during this early stage of life involve crucial cranial, subarachnoid, cerebrospinal fluid dynamics, and brain-specific anatomical and pathophysiological characteristics (Kolb et al., 2014). Therefore, undergoing anesthesia and surgery between the ages of 0 and 3 years is recognized as a significant event that could potentially result in abnormalities in neurocognitive development and brain function.

A large amount of preclinical evidence suggests that the developing brain is vulnerable to stressors such as anesthetic drugs and surgery, with effects that are highly dependent on age and drug dosage. Specifically, earlier exposure and higher doses are associated with more severe consequences. However, clinical studies investigating the neurotoxic effects of anesthesia and surgery have yielded inconsistent results. Many of these studies have focused on assessing cognitive performance in young children shortly after anesthesia exposure, typically during hospitalization or within 7 days thereafter. Recent research indicates no consistent significant impacts of anesthesia on general intelligence, memory, or language abilities (Clausen et al., 2018; Ing et al., 2022). A systematic review encompassing 13 retrospective studies highlighted that exposure to general anesthesia before age three may moderately increase the risk of neurodevelopmental disorders (Zhang et al., 2015). In a case–control study involving 47 infants and toddlers aged 1–36 months undergoing noncardiac surgery, no significant differences in overall cognitive performance were observed (Waitayawinyu et al., 2023). Notably, inhalation anesthetics, commonly used as primary agents in pediatric general anesthesia, have been implicated in cognitive effects such as postoperative delirium (Fan et al., 2018). General anesthesia can also induce delirium upon awakening and postoperative behavioral abnormalities among preschoolers (Kim et al., 2021). While short-term observation post-anesthesia/surgery can enhance case monitoring efficiency, findings may be confounded by factors such as hospitalization-related pain, anxiety, perioperative medications like analgesics and sedative-hypnotics, and other neurocognitive disorders (Kolb et al., 2014; Cusick et al., 2022). Additionally, nutritional status during hospitalization might influence children’s behavioral and cognitive performance, potentially contributing to the variability observed in study outcomes.

Extended observation periods effectively mitigate perioperative impacts on neurocognitive and behavioral performance in young children, prompting researchers’ heightened concern regarding the long-term consequences of early-life exposure to anesthesia and surgery. Recent studies indicate that early exposure to anesthesia and surgery does not correlate with subsequent intelligence and developmental outcomes, after controlling for age and genetic factors (Sun et al., 2016; O’Leary et al., 2019). Conversely, multiple studies suggest otherwise; for instance, anesthesia or surgery before the age of 4 is linked to reduced academic achievement and intellectual performance in later adolescence (16–18 years) (Glatz et al., 2017). Consequently, a definitive consensus regarding the enduring impacts of early anesthesia and surgical experiences remains elusive.

The perioperative period involves complex and stressful events that may impact brain and systemic functions. Factors such as surgical type/site, medication usage, awakening times, and hospitalization duration may influence the recovery of postoperative multiorgan function. It remains uncertain whether these factors affect behavioral and intellectual-cognitive development in the short or long term, given the current lack of evidence on their potential impact on developmental abnormalities in children. Consequently, this study aims to examine children who underwent anesthesia or surgery between 0 and 3 years of age to assess how this exposure affects their behavioral development and intellectual-cognitive performance at ages 4–6. Additionally, it seeks to investigate correlations between various factors—such as surgical site, anesthetic drugs, number of procedures, awakening time, and weight at exposure—and behavioral and cognitive outcomes. This study offers a chance to identify potential risk factors influencing cognitive and behavioral development in infants and young children exposed to surgical anesthesia between ages 0 and 3. Findings may inform the enhancement of perioperative care protocols tailored for children.

## 2 Methods and materials

### 2.1 Population

The study was approved by the Ethics Committee of the First Affiliated Hospital of Guangxi Medical University [2019 (KY-E-047)], and all procedures adhered to local legislation and institutional guidelines. Participants’ legal guardians/next of kin provided written informed consent, and the study was registered in the clinical trials registry (ChiCTR1900024598). Data were collected from electronic medical records of participants aged 0–3 years who underwent general anesthesia and surgery at the First Affiliated Hospital of Guangxi Medical University between 2012 and 2015. The study evaluates behavioral development and intellectual cognitive performance in these children at ages 4–6. Concurrently, age-, race-, and gender-matched children from the same region, who did not undergo general anesthesia or surgery, comprised the control group. Data collection and evaluation concluded in December 2021. Inclusion criteria for the anesthesia/surgery (AS) group included participants aged 0–3 years who underwent anesthesia and surgery, had ASA grades I-III, were currently aged 4–6 years, and whose guardians provided informed consent. Exclusion criteria comprised preterm infants (<36 weeks gestation), pre-existing developmental, cognitive, or behavioral abnormalities, neurological disorders, history of birth asphyxia, cardiac, thoracic, or neurosurgical procedures, emergency or malignant tumor surgeries, maternal anesthesia during gestation, communication difficulties of guardians, or mid-study withdrawals.

### 2.2 Cognitive and behavioral developmental assessment

Cognitive and behavioral development was evaluated using the Wechsler Preschool Primary Scale of Intelligence, Fourth Edition (WPPSI-IV), Gesell Development Schedules (GSCH), and Achenbach Children’s Behavior Checklist (CBCL). The WPPSI-IV assesses participants’ verbal, Visual–Spatial, Fluid Reasoning, Processing Speed, and Working Memory abilities. Full-Scale IQ (FSIQ), reflecting overall intelligence, was derived from normed scores of subtests. Additional indices evaluated include the Verbal Comprehension Index (VCI), Visual Spatial Index (VSI), Fluid Reasoning Index (FRI), Working Memory Index (WMI), and Processing Speed Index (PSI), offering a comprehensive view of cognitive function. The GSCH evaluates young children’s behavior and development across adaptive behavior, gross motor skills, fine motor skills, verbal ability, and personal-social behavior. It uses a model of typical child behavior norms (physical: standing, sitting, lying, walking, grasping; intellectual: visual perception, auditory perception, speech, and basic object analysis) as benchmarks for assessing motor, adaptive, verbal, and social abilities. Developmental age is inferred by comparing a child’s performance with these norms, calculating a Developmental Quotient (DQ) known for its objectivity and validity. The CBCL demonstrates strong validity in evaluating and predicting abnormalities in children’s personality and behavioral development, primarily for screening social skills and behavioral issues. Designed for children aged 4–16 years, it is completed anonymously by parents familiar with the child. Comprising 113 items, the CBCL assesses behaviors on a scale of 0, 1, and 2 (“not present,” “sometimes present,” and “often present,” respectively), reflecting the child’s behavior over the previous 2 months; higher scores indicate greater severity of behavioral problems.

### 2.3 Data collection

Participants’ baseline characteristics before surgery included age, gender, ethnicity, height, weight, and ASA status, along with previous surgical history. Perioperative details encompass surgical site, anesthetic drug usage, operation duration, recovery duration, and hospital stay length. The control group comprised children aged 4–6 from the same region, matching the inclusion criteria of the AS group, with recorded baseline characteristics of age, gender, ethnicity, height, and weight. Scores from the WPPSI-IV, GSCH, and CBCL scales were compared between the AS group and the control group to investigate the influence of early-life anesthesia/surgery experiences (0–3 years) on cognitive and behavioral development over the long term. Within the AS group, we examined how gender, ethnicity, weight, surgical site, operation duration, anesthetic drug use, recovery duration, hospital stay length, and previous surgical history influence behavioral development and cognitive performance. This exploration aims to identify factors potentially impacting children’s cognitive and behavioral outcomes following anesthesia/surgery.

### 2.4 Statistical analysis

SPSS 24.0 software (SPSS Inc., Chicago, United States) was applied for data analysis. The Kolmogorov–Smirnov test was used for normality and lognormality tests. Measurement data conforming to normal distribution were presented as mean ± standard deviation (mean ± SD), and categorical variables were presented as the number (percentage) of cases (*n*, %). Comparisons between two groups were performed by two independent samples *t*-tests; comparisons between more than two groups were analyzed by the one-way ANOVA method. Correlation analysis was performed by Spearman’s correlation analysis. *p* < 0.05 was set as statistically significant.

## 3 Results

### 3.1 Trial enrollment and characteristics comparison

**Figure 1:**
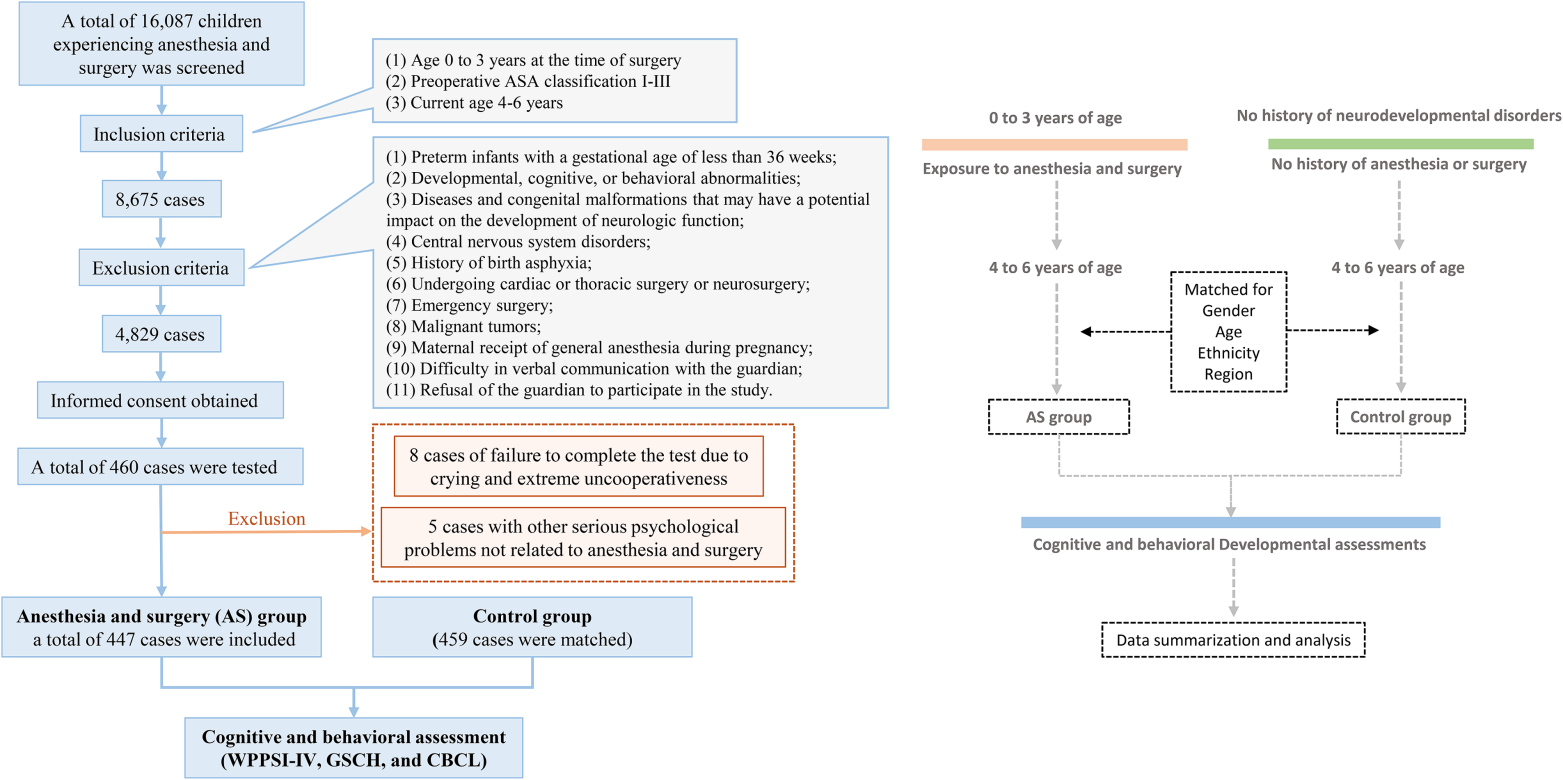
Flow chart of the trial.

A total of 16,087 children underwent surgical procedures at the First Affiliated Hospital of Guangxi Medical University between 2012 and 2015. Following the inclusion criteria, 8,675 cases were deemed eligible. By December 2021, 4,829 cases were contacted by telephone to participate in the study, resulting in 460 children agreeing to undergo testing. Of these, eight cases were unable to complete the tests due to crying and extreme uncooperativeness, while five cases were excluded due to severe unrelated psychological issues; these children were referred to appropriate specialists for further diagnosis and treatment. The remaining 447 cases were enrolled in the AS (anesthesia/surgery) group. Additionally, 459 children aged 4–6 years, matched for ethnicity and gender, who had not undergone general anesthesia or surgery in the same region, were enrolled as the Control group (Figure 1). Table 1 presents a comparison of the characteristics of both groups.

**Table 1:** Comparison of basic characteristics between AS group and control group.

Item	Group	
	Control group (*n* = 459)	AS group (*n* = 447)
Gender		
Male (*n*, %)	309 (67.3)	340 (76.1)
Female (*n*, %)	150 (32.7)	107 (23.9)
Ethnic group		
Han (*n*, %)	235 (51.2)	217 (48.5)
Other (*n*, %)	224 (48.8)	230 (51.5)
Age		
(4.0 ~ 5.0) (*n*, %)	206 (44.9)	200 (44.7)
(5.0 ~ 6.11) (*n*, %)	253 (55.1)	247 (55.3)
Surgical site		
Head and face (*n*, %)	–	116 (26.0)
Epigastrium (*n*, %)	–	31 (7.0)
Hypogastrium (*n*, %)	–	179 (40.0)
Perineum (*n*, %)	–	59 (13.2)
Limbs (*n*, %)	–	62 (13.8)
Age at the time of surgery (year)		
0–0.5 (*n*, %)	–	108 (24.2)
0.5–1 (*n*, %)	–	129 (28.9)
1–2 (*n*, %)	–	60 (13.4)
2–3 (*n*, %)	–	150 (33.5)
Weight at the time of surgery (kg)		
<5 (*n*, %)	–	39 (8.7)
5 ~ 10 (*n*, %)	–	232 (51.9)
10 ~ 15 (*n*, %)	–	138 (30.9)
>15 (*n*, %)	–	38 (8.5)
Length of surgery (h)		
<1 (*n*, %)	–	217 (48.5)
1 ~ 2 (*n*, %)	–	176 (39.4)
>2 (*n*, %)	–	54 (12.1)
Length of recovery (min)		
<30 (*n*, %)	–	262 (58.6)
30 ~ 60 (*n*, %)	–	134 (30.0)
>60 (*n*, %)	–	51 (11.4)
Number of surgeries experienced (times)		
1 (*n*, %)	–	408 (91.3)
2 (*n*, %)	–	33 (7.4)
>2 (*n*, %)	–	6 (1.3)

The AS group was stratified by ethnicity into Han and other ethnic groups, by gender into female and male groups, by age at the time of surgery into 1–5 months, 6–12 months, 1–2 years, and 2–3 years groups, by body weight at the time of surgery into <5 kg, 5–10 kg, 10–15 kg, and > 15 kg groups, by number of surgeries experienced into 1-time, 2-times, and > 2-times groups, by surgical site into head and face surgery, upper abdominal surgery, lower abdominal surgery, perineal surgery, and limbs surgery groups, by length of surgery into <1 h, 1–2 h, and > 2 h groups, and by time of awakening into <30 min, 30–60 min, and > 60 min groups (Table 1).

### 3.2 Effects of anesthesia and surgery on cognitive and behavioral development

**Figure 2:**
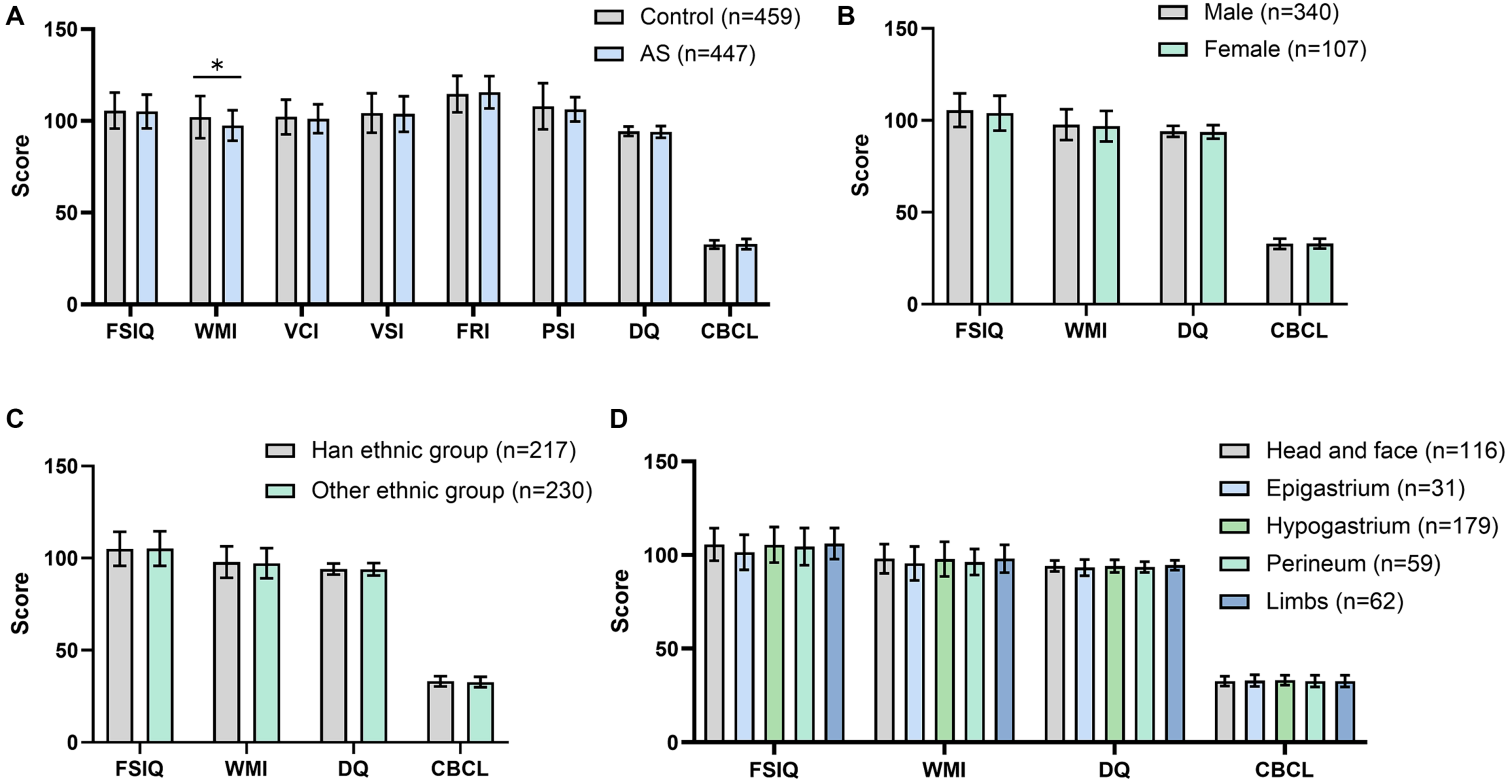
Effects of anesthesia/surgery on cognitive and behavioral development and subgroup analysis. **(A)** Comparison of cognitive and behavioral developmental scores between children in the AS group and control group; **(B)** Subgroup analysis of the AS group: comparison of postoperative cognitive and behavioral developmental indices (FSIQ, WMI, DQ, and CBCL) in children of different genders; **(C)** Subgroup analysis of the AS group: comparison of postoperative FSIQ, WMI, DQ, and CBCL in children of different ethnicities; **(D)** Subgroup analysis of the AS group: comparison of postoperative FSIQ, WMI, DQ, and CBCL in children in different sites of surgery. FSIQ, full Scale IQ; WMI, working memory index; DQ, developmental quotient; CBCL, child behavior checklist. Comparisons between two groups were performed by two independent samples *t*-tests; comparisons between more than two groups were analyzed by the one-way ANOVA method. *, *p* < 0.05.

The WMI score of the AS group (97.51 ± 8.35) was significantly lower than that of the Control group (102.03 ± 11.52) (*p* < 0.05) (Figure 2A). There was no statistically significant difference in the FSIQ, VCI, VSI, FRI, PSI, DQ score, and CBCL score of the AS group when compared with the Control group (*p* > 0.05) (Figure 2A).

### 3.3 Effects of gender, ethnicity, and surgical site on postoperative cognitive and behavioral development

Comparisons within the AS group revealed no statistical differences in FSIQ, WMI, DQ, and CBCL scores between children of different genders (Figure 2B), ethnicities (Figure 2C), and surgical sites (Figure 2D).

### 3.4 Age and weight correlate with postoperative cognitive performance and behavioral development

**Figure 3:**
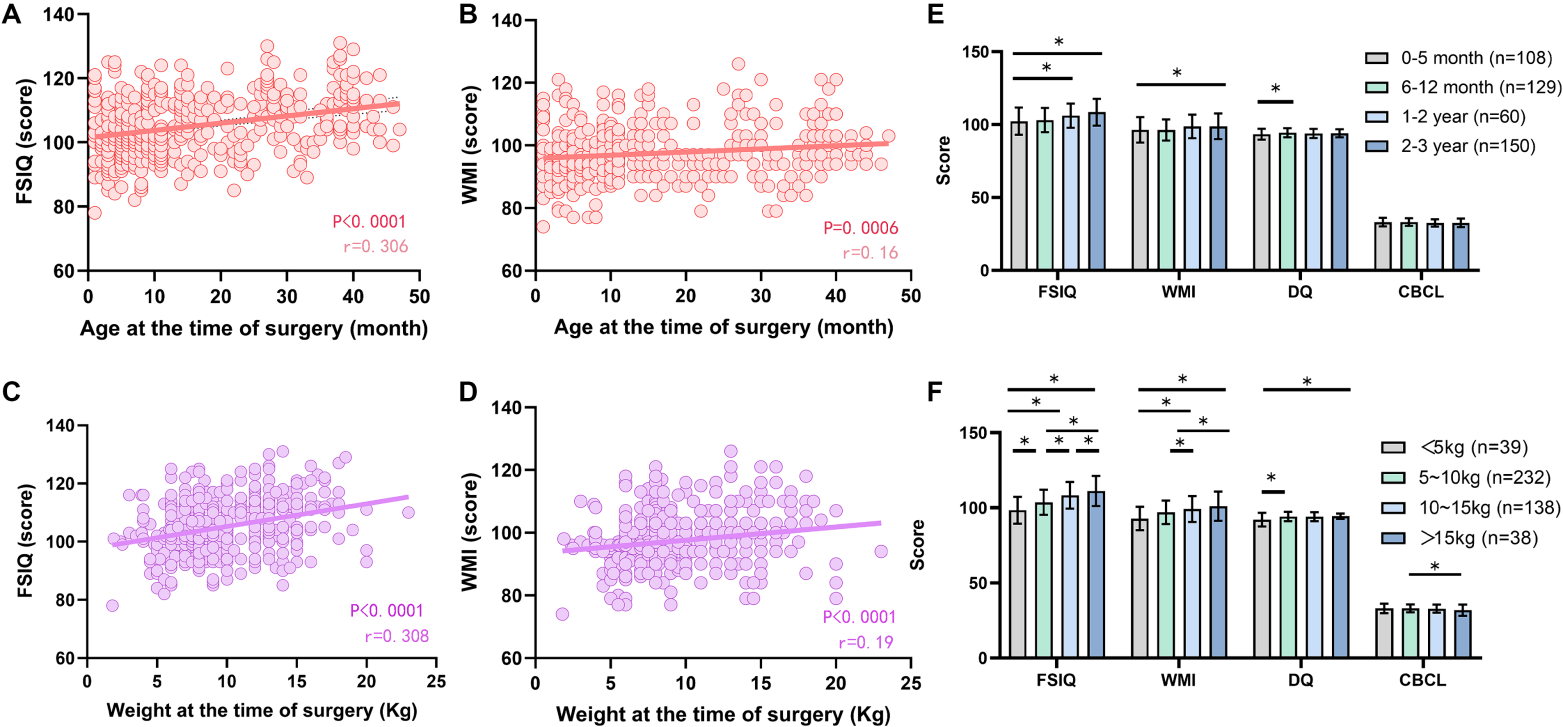
Effect of age and body weight at the time of surgery on postoperative cognitive and behavioral development. **(A)** Correlation analysis between FSIQ scores and age; **(B)** Correlation analysis between WMI scores and age; **(C)** Comparison of postoperative FSIQ, WMI, DQ, and CBCL in children of different ages; **(D)** Correlation analysis between FSIQ scores and body weight; **(E)** Correlation analysis of WMI scores and body weight; **(F)** Comparison of postoperative FSIQ, WMI, DQ, and CBCL in children of different body weights. FSIQ, full Scale IQ; WMI, working memory index; DQ, developmental quotient; CBCL, child behavior checklist. Spearman’s analysis was used for correlation analysis, and the one-way ANOVA method was used for comparison among multiple groups. *, *p* < 0.05.

Both age and weight at the time of surgery were significantly and positively correlated with FSIQ and WMI scores (Figures 3A–D), and no statistically significant correlation was obtained with either DQ or CBCL scores (Supplementary Figures S1A–D). The DQ values of children aged 6–12 months at the time of surgery were higher than those of the 0–5 month group (*p* < 0.05); the FSIQ of children in the 1–2 years old group was higher than that of the 0–5 month group (*p* < 0.05); and the FSIQ and WMI of children in the 2–3 years old group were higher than those of children in the 0–5 month group (*p* < 0.05). The difference in scores between the remaining groups was not statistically significant (*p* > 0.05) (Figure 3E). FSIQ, WMI, and DQ scores were higher in the 5 ~ 10 kg group than in the <5 kg group (*p* < 0.05), and the difference in CBCL scores between the two groups was not statistically significant (*p* > 0.05). The FSIQ and WMI were higher in the 10 ~ 15 kg group than in the <5 kg group (*p* < 0.05), and the difference in DQ and CBCL scores between the two groups was not statistically significant (*p* > 0.05). Comparing the 10–15 kg group with the 5–10 kg group, FSIQ and WMI were higher in the former group (*p* < 0.05), and the differences in DQ and CBCL scores were not statistically significant (*p* > 0.05). Comparing the >15 kg group with the <5 kg group, the former had higher FSIQ, WMI, and DQ scores (*p* < 0.05), and the difference in CBCL scores was not statistically significant (*p* > 0.05). The FSIQ and WMI were higher in the >15 kg group than in the 5–10 kg group (*p* < 0.05), the CBCL score was lower (*p* < 0.05), and the difference in DQ scores between the two groups was not statistically significant (*p* > 0.05). The FSIQ score was higher in the >15 kg group than in the 10–15 kg group (*p* < 0.05), and the differences in WMI, DQ, and CBCL scores were not statistically significant (*p* > 0.05) (Figure 3F).

### 3.5 Effects of different anesthetics on postoperative cognitive and behavioral development

**Figure 4:**
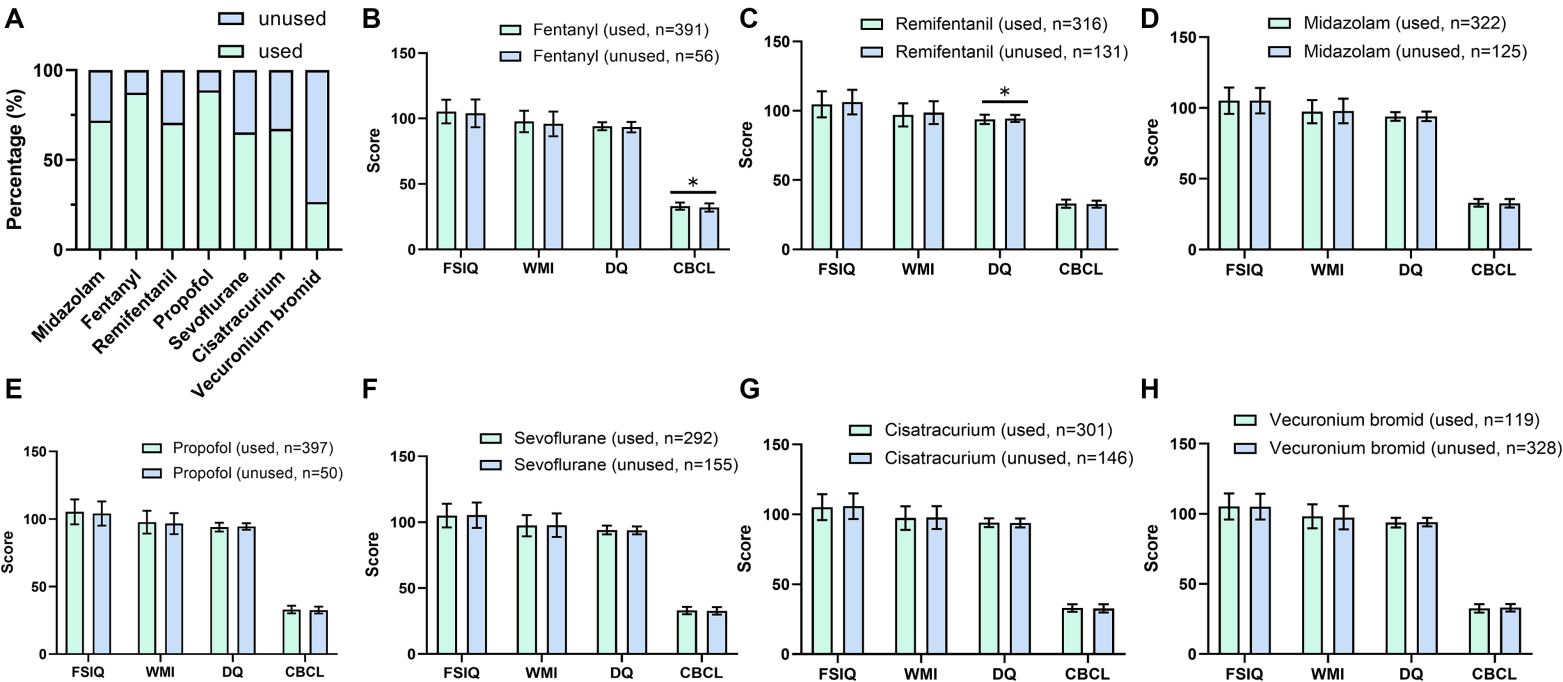
Effects of different anesthetics on postoperative cognitive and behavioral development. **(A)** The use of different anesthetic drugs; **(B)** The effect of fentanyl on postoperative FSIQ, WMI, DQ, and CBCL; **(C)** The effect of remifentanil on postoperative FSIQ, WMI, DQ, and CBCL; **(D)** The effect of midazolam on postoperative FSIQ, WMI, DQ, and CBCL; **(E)** The effect of propofol on postoperative FSIQ, WMI, DQ, and CBCL; **(F)** The effect of sevoflurane on postoperative FSIQ, WMI, DQ, and CBCL; **(G)** The effect of cis-atracurium on postoperative FSIQ, WMI, DQ, and CBCL; and **(H)** The effect of vecuronium bromide on postoperative FSIQ, WMI, DQ, and CBCL. FSIQ, full Scale IQ; WMI, working memory index; DQ, developmental quotient; CBCL, child behavior checklist. Comparisons between multiple groups were analyzed by one-way ANOVA analysis. *, *p* < 0.05.

Seven anesthetic drugs, including midazolam, fentanyl, remifentanil, propofol, sevoflurane, cis-atracurium, and vecuronium bromide, were selected to investigate their impact on cognitive and behavioral development in children. The analysis encompassed 447 cases (AS group), with each drug’s usage evaluated separately. Participants were divided into “Used” and “Unused” groups for each medication. Figure 4A illustrates the distribution of anesthetic usage across the study cohort. The CBCL scores were elevated in children using fentanyl (33.04 ± 2.73 & 32.02 ± 3.12, *p* < 0.05, Figure 4B). Children treated with remifentanil had lower DQ values (93.82 ± 3.38 & 94.49 ± 2.58, *p* < 0.05, Figure 4C). The use of other anesthetics did not yield statistically significant differences in the children’s behavioral and cognitive development in the postoperative period (Figures 4D–H).

### 3.6 Length of surgery and recovery time correlate with postoperative cognitive performance

**Figure 5:**
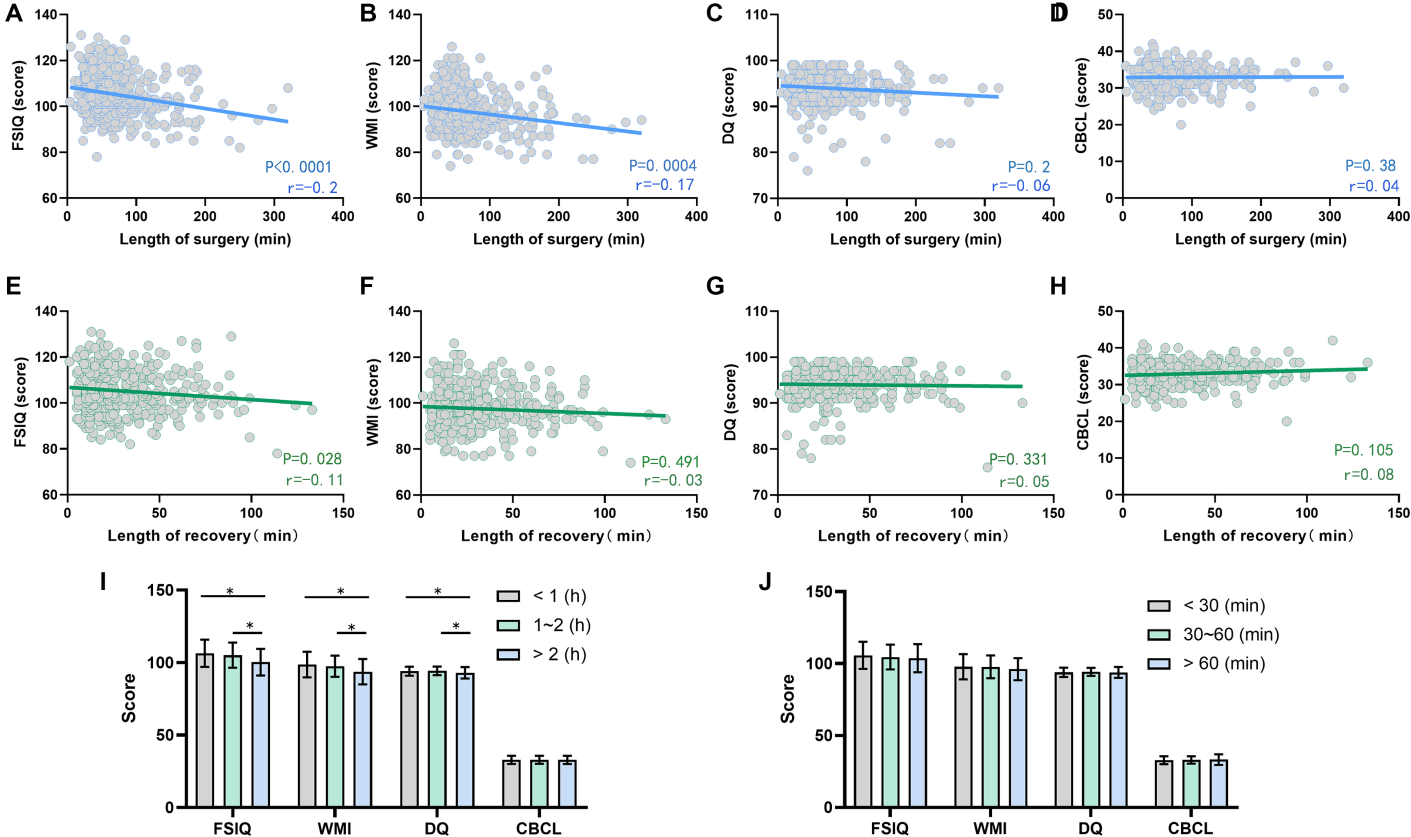
Effects of operation length and awakening time on postoperative cognitive and behavioral development. **(A–D)** Correlation analysis of surgery duration with FSIQ score **(A)**, WMI **(B)**, DQ **(C)**, and CBCL score **(D)**; **(E–H)** Correlation analysis of recovery time with FSIQ score **(E)**, WMI **(F)**, DQ **(G)**, and CBCL score **(H)**; **(I)** The effect of different surgical durations on the children’s postoperative FSIQ, WMI, DQ, and CBCL; **(J)** Comparison of postoperative FSIQ, WMI, DQ, and CBCL in children with different recovery times. FSIQ, full Scale IQ; WMI, working memory index; DQ, developmental quotient; CBCL, child behavior checklist. Spearman’s analysis was used for correlation analysis, and the one-way ANOVA method was used for comparison among multiple groups. *, *p* < 0.05.

Both the length of surgery and recovery time were negatively correlated with the FSIQ score (*p* < 0.05). Length of surgery was negatively correlated with WMI scores (*p* < 0.05). There was no significant correlation between length of surgery with both DQ and CBCL, and recovery time with WMI, DQ, and CBCL scores (Figures 5A–H).

Comparing the surgery duration >2 h group with the <1 h group, the former had lower FSIQ, WMI, and DQ scores (*p* < 0.05), and the difference in CBCL scores was not statistically significant (*p* > 0.05). The FSIQ, WMI, and DQ values were lower in the surgery duration >2 h group compared with the 1 ~ 2 h group (*p* < 0.05), and the difference in CBCL scores between the two groups was not statistically significant (*p* > 0.05). The difference in each index was not statistically significant when the 1 ~ 2 h group was compared with the <1 h group (*p* > 0.05, Figure 5I). There were no statistically significant differences in cognitive and behavioral developmental scores between groups of different recovery times (*p* > 0.05, Figure 5J).

### 3.7 Increased number of surgeries experienced early in life can lead to impaired cognitive and behavioral development

**Figure 6:**
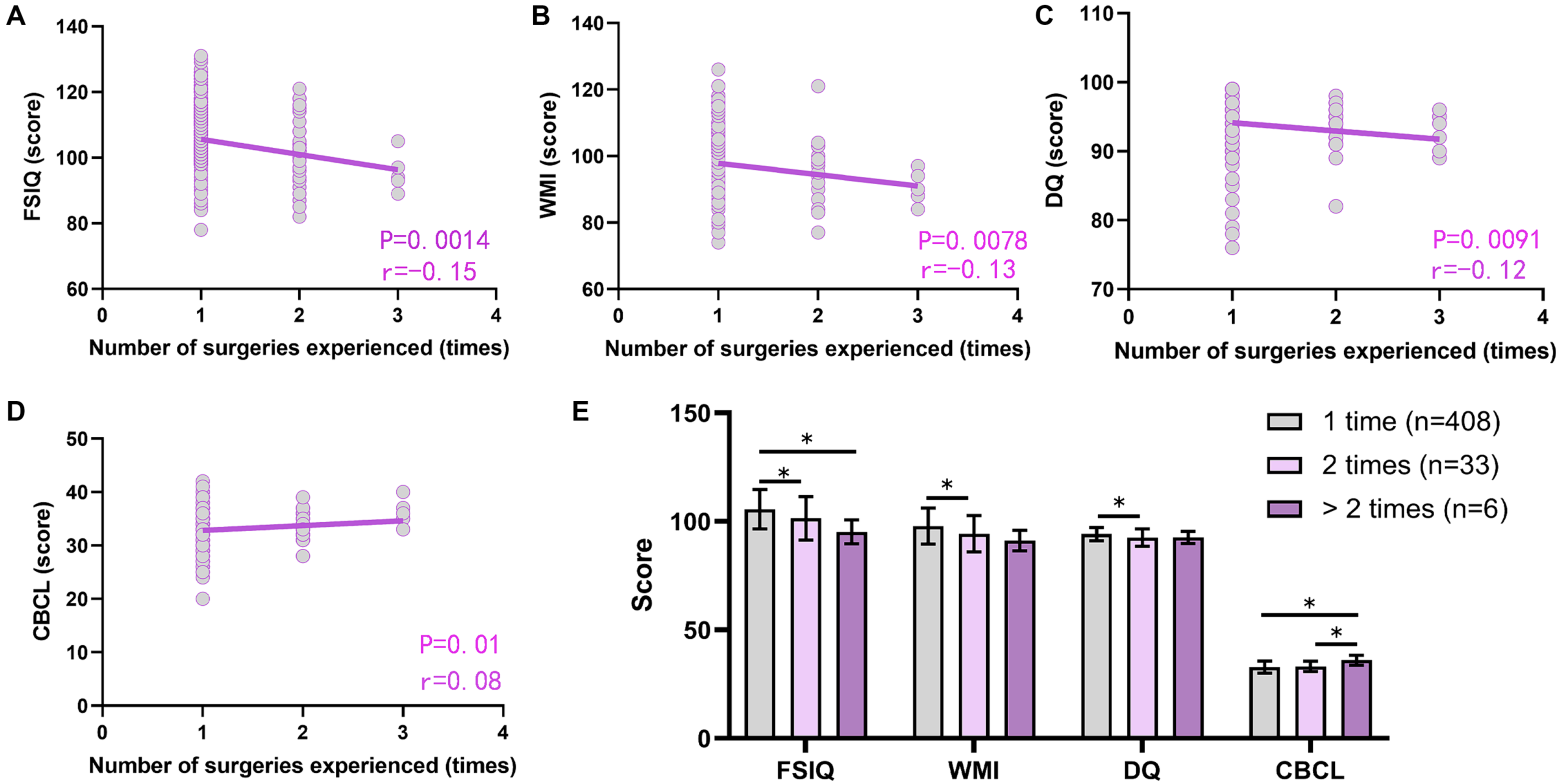
Effect of times of anesthesia/surgery experienced on postoperative cognitive and behavioral development. **(A–D)** Correlation analysis of the times of anesthesia/surgery experienced with FSIQ **(A)**, WMI **(B)**, DQ **(C)**, and CBCL **(D)** scores; **(E)** Comparison of postoperative FSIQ, WMI, DQ, and CBCL in children who underwent different times of anesthesia/surgery. FSIQ, full Scale IQ; WMI, working memory index; DQ, developmental quotient; CBCL, child behavior checklist. Spearman’s analysis was used for correlation analysis, and the one-way ANOVA method was used for comparison among multiple groups. *, *p* < 0.05.

The number of surgeries experienced had a significant negative correlation with FSIQ, WMI, and DQ (*p* < 0.05, Figures 6A–C) and a positive correlation with CBCL (*p* > 0.05, Figure 6D). Comparing the number of surgeries experienced 2 times with a single procedure at 0–3 years of age, the former showed lower scores of FSIQ, WMI, and DQ scores (*p* < 0.05), and the difference in CBCL scores was not statistically significant (*p* > 0.05). Comparing the number of surgeries experienced >2 times with a single occasion, the former had lower FSIQ (*p* < 0.05) and higher CBCL scores (*p* < 0.05), and the difference in WMI and DQ scores was not statistically significant (*p* > 0.05). Experiencing >2 surgeries compared with 2-time occasions, the former had a higher CBCL score (*p* < 0.05), and the difference in FSIQ, WMI, and DQ scores was not statistically significant (*p* > 0.05, Figure 6E).

## 4 Discussion

Each year, millions of children undergo medical procedures requiring anesthesia, primarily employing general anesthesia for pediatric surgeries. Commonly used anesthetic agents such as sevoflurane, midazolam, propofol, and fentanyl are recognized for their neurotoxic potential, including neuronal cell death induction, synaptic dysfunction, and impaired energy metabolism, which can lead to behavioral and cognitive abnormalities (Xu et al., 2019; Diana et al., 2020; Zhang and Li, 2023; Zhou et al., 2024). Animal studies have demonstrated that exposure to general anesthesia may promote programmed neuronal cell death early in life, resulting in impaired learning and behavioral functions (Jevtovic-Todorovic and Useinovic, 2023; Kim et al., 2023). For instance, Jevtovic-Todorovic et al. (2003) observed apoptotic changes in brain neurons of 7-day-old rats exposed to imipramine, nitrous oxide, and isoflurane for 6 h, with inhibited induction of hippocampal long-term potentiation (LTP). Long-term behavioral and cognitive impairments persisted into adulthood in rats exposed to anesthesia during the neonatal period, indicating potential long-lasting effects on brain development (Gentry et al., 2013). Kong et al. (2012) demonstrated that maternal inhalation of 3% isoflurane for 1 h in neonatal rats resulted in impaired learning and memory, increased caspase-3 activity, and disrupted synaptic structures. Human cohort studies investigating the neurodevelopmental impacts of early-life anesthesia and surgery have yielded inconsistent conclusions due to variations in case inclusion/exclusion criteria and inherent biases (O'Leary, 2019). A study focusing on preschool children, particularly infants and those aged 0–3 years, found associations between surgery/anesthesia exposure and impaired social competence development from 0 to 6 years of age (Waitayawinyu et al., 2023).

In the present study, we found that infants and children aged 0–3 years who underwent general anesthesia and surgery had lower WMI scores at 4–6 years of age compared to normal children, while no significant differences were observed in FSlQ, VCI, VSI, FRI, PSI, DQ, and CBCL scores. WMI is commonly used to assess the breadth and speed of processing information during cognitive activities. Decreased WMI reflects deficits in attention, concentration control, and reasoning (Anker et al., 2022). Our findings suggest that anesthesia and surgery have affected the efficiency of children in performing cognitive activities. Despite the importance of FSIQ as an indicator of cognitive and intellectual development in children, it has been shown in similar studies that the effects of anesthesia and surgery on FSIQ in children are not significant. For example, dental treatment under sevoflurane, nitrous oxide, and propofol anesthesia did not result in significant differences in preoperative and postoperative FSlQ comparisons in 4- to 6-year-old children (Zhou et al., 2021). In a randomized controlled trial of 447 infants at 60 weeks undergoing inguinal hernia repair under awake regional anesthetics or sevoflurane-based general anesthesia, the average FSIQ score was 99.08 (SD, 18.35) in the awake regional anesthesia group and 98.97 (SD, 19.66) in the general anesthesia group, suggesting that exposure to general anesthesia early in life for a relatively restricted period does not alter postoperative neurodevelopmental outcomes (McCann et al., 2019).

We analyzed 447 children who underwent anesthesia and surgery, examining factors such as ethnicity, age, weight, surgical site, surgery duration, awakening time, anesthetic medications, and number of surgeries. Our findings indicate that gender, ethnicity, and surgical site did not significantly affect cognitive and behavioral outcomes post-surgery and anesthesia. However, younger age and lower body weight at the time of anesthesia and surgery correlated with later impaired cognitive and behavioral development. This association may stem from the timing of exposure during critical stages of brain development. Previous research has linked anesthesia exposure during the rapid brain development period (between 28 weeks and 24 months) with subsequent neurodevelopmental deficits (Houck and Vinson, 2017). Another cohort study highlighted reduced academic and cognitive performance in adolescents who underwent anesthesia and surgery before age 4, suggesting heightened risks with younger age at exposure (Naumann et al., 2012).

We also investigated the impact of various anesthetic drugs on children’s postoperative cognition and behavior. The commonly used drugs included midazolam, fentanyl, remifentanil, propofol, sevoflurane, cis-atracurium, and vecuronium bromide. Our findings revealed that fentanyl administration was associated with elevated CBCL behavioral abnormality scores, while children administered remifentanil exhibited lower postoperative GSCH scores (DQ) compared to non-users. This observation is intriguing as both traditional long-acting and ultrashort-acting fentanyl analgesics may contribute to impaired cognitive and behavioral development. Animal studies, using zebrafish as a model, suggest potential adverse effects of opioids on neurodevelopment and behavior. For instance, exposure to a 5 mg/L fentanyl solution induced heightened anxiety, reduced aggression, and behavioral sensitization in zebrafish larvae. Furthermore, embryonic exposure to fentanyl resulted in significant alterations in neural activity and neuronal plasticity (Wang et al., 2022). *In vitro* studies with isolated neuronal cells exposed to fentanyl and remifentanil indicated increased neuroinflammation and oxidative stress, with remifentanil showing more pronounced neuroinflammatory effects (Taghizadehghalehjoughi et al., 2024). Moreover, research by Jantzie et al. (2020) demonstrated persistent peripheral inflammatory hyperreactivity in perinatally methadone-exposed young rats, suggesting a potential exacerbation of brain damage secondary to opioid exposure and increased susceptibility to neuroinflammation and neurological disorders. Studies on perinatally methadone-exposed rats have shown MRI-documented cerebral white matter microstructural damage, neuroimmune dysfunction, and cognitive deficits, alongside systemic inflammatory response syndromes and persistent brain inflammation. These findings suggest alterations in the central nervous system microenvironment, disruption of neurodevelopmental homeostasis, and concurrent neurological injury. Similar effects have been observed in offspring born to mothers administered methadone, potentially resulting in long-term impairments in associative learning, executive control, and an increased likelihood of behavioral and attention deficits (Lotsch, 2005; Franchi et al., 2019). Both methadone and remifentanil act as μ-opioid receptor agonists and appear to have analogous effects on neurological development. Our study’s results indicate that the use of remifentanil decreased Gesell test DQ scores in children, suggesting poorer gross and fine motor development compared to typical children. This outcome may be linked to remifentanil-triggering mechanisms akin to those observed with methadone in neurodevelopment.

While we have not observed an association between some anesthetic drugs and impaired cognitive and behavioral development, an increasing body of evidence highlights the adverse effects of various anesthetic agents such as midazolam, propofol, and sevoflurane on the physiological functioning of nerve cells. A study involving 841 children exposed to general anesthetics found that anesthesia exposure was associated with significantly higher scores on CBCL questions, indicating more pronounced internalizing behavioral problems, though exposure did not affect Full-Scale IQ (FSIQ) scores (Ing et al., 2021). Another study involving 147 very preterm infants, with a median gestational age of 27.7 weeks, suggested that early exposure to midazolam may adversely affect hippocampal growth and development, with reductions in hippocampal volume correlating negatively with midazolam dosage and leading to reduced cognitive performance at a corrected age of 18.7 months (Duerden et al., 2016). Multiple studies have also provided evidence implicating propofol in developmental neurotoxicity, characterized by neuronal and mitochondrial apoptosis, oxidative stress, and inhibition of the electron transport chain (He et al., 2020; Liang et al., 2022; She et al., 2023). It is important to note, however, that the doses used in animal or *in vitro* studies are often much higher than those used clinically. Clinical practice typically involves combination regimens aimed at minimizing the dose of any single drug to mitigate potential adverse effects, tailored to the specific surgical and physiological needs of the child. Despite efforts to minimize drug-related adverse effects in clinical settings, the potential impact of anesthetic drugs on neuronal cell function remains a concern. Prolonged surgery and recovery times, as well as multiple early-life surgeries, have been identified as potential factors contributing to impaired intellectual and behavioral development in children. Previous research supports this, indicating that children exposed to general anesthesia and multiple surgeries may face long-term risks related to processing speed, fine motor function, and behavioral assessment, especially following repeated anesthesia exposures (O'Leary, 2019). For instance, Warner et al. (2021) reported decreased scores in fine motor skills and processing speed among children with multiple anesthesia exposures, underscoring concerns regarding developmental neurotoxicity associated with anesthetic drugs.

This study has several limitations. We retrospectively recorded anesthesia/surgical exposure data and prospectively evaluated children’s neurocognition and behavior. One of our main purposes was to observe the effects of experiencing anesthesia and surgery early in life on later neurocognitive and behavioral development. Our study design lacked strict control over the timing of exposure and outcome assessments, leading to variability in the age at which children in the anesthesia-surgery group were exposed and assessed post-exposure. Although we controlled for the risk of confounding by matching controls for age, sex, and region, however, as an observational study, the ability to infer causality is limited, and the retrospective study design introduces a risk of bias and unmeasured sources of confounding from other factors that cannot be fully mitigated by the use of matching. Additionally, surgery duration correlated with disease severity, surgical complexity, and surgeon expertise, affecting anesthesia exposure duration. We did not quantitatively compare disease severity among children who underwent surgery. Prolonged surgery or anesthesia durations imply higher anesthetic doses and fluid loss, potentially impacting awakening times. Future studies should explore how varying drug doses and intraoperative factors like bleeding and fluid management influence postoperative cognitive and behavioral outcomes. Furthermore, our study did not incorporate brain imaging data (MRI or CT) due to its retrospective collection of perioperative data from non-cranial surgeries, as brain imaging is not routine for most children undergoing surgery.

This study offers insights into the potential adverse effects of surgical anesthesia exposure in infants and young children on postoperative cognitive and behavioral development. However, it remains uncertain whether these effects persist, amplify, or lead to more serious consequences over the longer term.

## 5 Conclusion

This study investigated the impact of surgery and anesthesia exposure on cognitive and behavioral development in infants and young children aged 0–3 years. The findings indicate that higher frequency of exposure to surgeries and anesthesia, younger age at exposure, and lower body weight all have negative effects on cognitive and behavioral development. Furthermore, opioid analgesics potentially affect behavior and cognition in young children, suggesting a need for cautious use of opioid analgesics like remifentanil and fentanyl.

## Data availability statement

The original contributions presented in the study are included in the article/Supplementary material, further inquiries can be directed to the corresponding authors.

## Ethics statement

The studies involving humans were approved by the Ethics Committee of the First Affiliated Hospital of Guangxi Medical University. The studies were conducted in accordance with the local legislation and institutional requirements. Written informed consent for participation in this study was provided by the participants’ legal guardians/next of kin.

## Author contributions

XJ: Conceptualization, Data curation, Formal analysis, Methodology, Software, Visualization, Writing – original draft, Writing – review & editing, Funding acquisition. ST: Conceptualization, Data curation, Formal analysis, Methodology, Software, Visualization, Writing – original draft, Writing – review & editing. YQ: Conceptualization, Data curation, Formal analysis, Methodology, Software, Visualization, Writing – original draft, Writing – review & editing. YW: Data curation, Formal analysis, Methodology, Software, Writing – review & editing. YJ: Data curation, Formal analysis, Methodology, Software, Writing – review & editing. SP: Data curation, Formal analysis, Methodology, Software, Writing – review & editing. CL: Data curation, Formal analysis, Methodology, Software, Writing – review & editing. JC: Conceptualization, Data curation, Project administration, Supervision, Writing – review & editing. TL: Conceptualization, Data curation, Project administration, Supervision, Writing – review & editing. YX: Conceptualization, Data curation, Funding acquisition, Project administration, Supervision, Writing – review & editing.
