# Supplementary material for: Experiencing anesthesia and surgery early in life impairs cognitive and behavioral development
Source: Front Neurosci. 2024 Jul 24;18:1406172. doi: 10.3389/fnins.2024.1406172 (PMC11303152; doi:10.3389/fnins.2024.1406172)
Supplement: Supplementary file 1 [file Data_Sheet_1.docx]

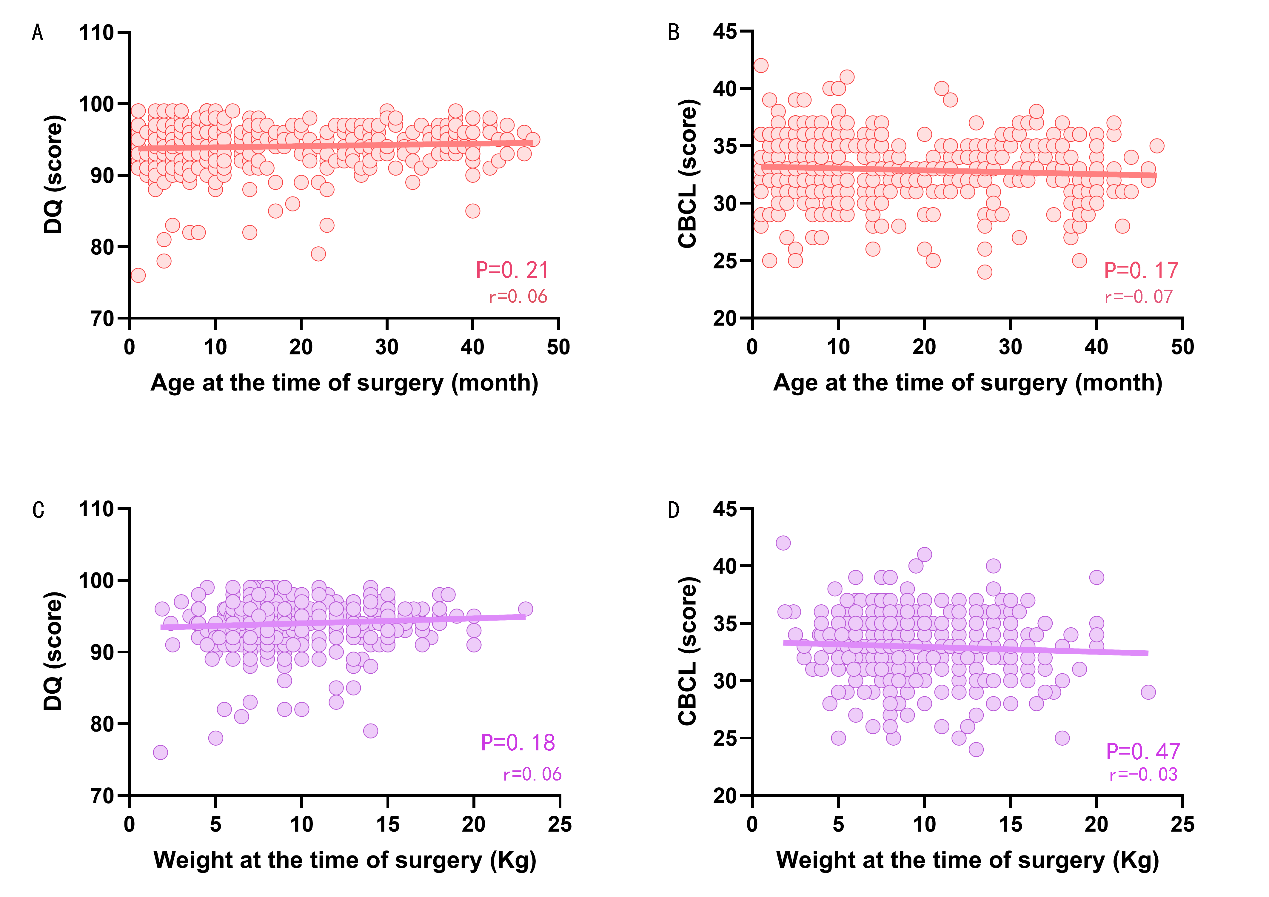


**Figure S1 Correlation analysis of age and body weight with postoperative cognitive and behavioral development.** (A) Correlation analysis between age and DQ scores; (B) Correlation analysis between age and CBCL scores; (C) Correlation analysis between weight and DQ; (D) Correlation analysis between weight and CBCL scores. DQ, developmental quotient; CBCL, child behavior checklist. Spearman's analysis was used for correlation analysis.
